# Neurofilament Light Regulates Axon Caliber, Synaptic Activity, and Organelle Trafficking in Cultured Human Motor Neurons

**DOI:** 10.3389/fcell.2021.820105

**Published:** 2022-02-14

**Authors:** Markus T. Sainio, Tiina Rasila, Svetlana M. Molchanova, Julius Järvilehto, Rubén Torregrosa-Muñumer, Sandra Harjuhaahto, Jana Pennonen, Nadine Huber, Sanna-Kaisa Herukka, Annakaisa Haapasalo, Henrik Zetterberg, Tomi Taira, Johanna Palmio, Emil Ylikallio, Henna Tyynismaa

**Affiliations:** ^1^ Stem Cells and Metabolism Research Program, Faculty of Medicine, University of Helsinki, Helsinki, Finland; ^2^ Molecular and Integrative Biosciences Research Program, Faculty of Biological and Environmental Sciences, University of Helsinki, Helsinki, Finland; ^3^ A.I. Virtanen Institute for Molecular Sciences, University of Eastern Finland, Kuopio, Finland; ^4^ Department of Neurology, Kuopio University Hospital, Kuopio, Finland; ^5^ Neurology, Institute of Clinical Medicine, University of Eastern Finland, Kuopio, Finland; ^6^ Clinical Neurochemistry Laboratory, Sahlgrenska University Hospital, Mölndal, Sweden; ^7^ Department of Psychiatry and Neurochemistry, Institute of Neuroscience and Physiology, The Sahlgrenska Academy at the University of Gothenburg, Mölndal, Sweden; ^8^ Department of Neurodegenerative Disease, UCL Institute of Neurology, London, United Kingdom; ^9^ UK Dementia Research Institute at UCL, London, United Kingdom; ^10^ Hong Kong Center for Neurodegenerative Diseases, Hong Kong, Hong Kong SAR, China; ^11^ Department of Veterinary Biosciences, Faculty of Veterinary Medicine, Department of Veterinary Biosciences for Electrophysiology, University of Helsinki, Helsinki, Finland; ^12^ Neuroscience Center, Helsinki Institute of Life Science, University of Helsinki, Helsinki, Finland; ^13^ Neuromuscular Research Center, Tampere University Hospital and Tampere University, Tampere, Finland; ^14^ Clinical Neurosciences, Neurology, University of Helsinki and Helsinki University Hospital, Helsinki, Finland; ^15^ Department of Medical and Clinical Genetics, University of Helsinki, Helsinki, Finland

**Keywords:** neurofilament light (NfL), motor neurodegeneration, axon, Charcot-Marie-Tooth (CMT) disease, induced pluripotent stem cells, motor neuron (MN)

## Abstract

Neurofilament light (NFL) is one of the proteins forming multimeric neuron-specific intermediate filaments, neurofilaments, which fill the axonal cytoplasm, establish caliber growth, and provide structural support. Dominant missense mutations and recessive nonsense mutations in the neurofilament light gene (*NEFL*) are among the causes of Charcot–Marie–Tooth (CMT) neuropathy, which affects the peripheral nerves with the longest axons. We previously demonstrated that a neuropathy-causing homozygous nonsense mutation in *NEFL* led to the absence of NFL in patient-specific neurons. To understand the disease-causing mechanisms, we investigate here the functional effects of NFL loss in human motor neurons differentiated from induced pluripotent stem cells (iPSC). We used genome editing to generate *NEFL* knockouts and compared them to patient-specific nonsense mutants and isogenic controls. iPSC lacking NFL differentiated efficiently into motor neurons with normal axon growth and regrowth after mechanical axotomy and contained neurofilaments. Electrophysiological analysis revealed that motor neurons without NFL fired spontaneous and evoked action potentials with similar characteristics as controls. However, we found that, in the absence of NFL, human motor neurons 1) had reduced axonal caliber, 2) the amplitude of miniature excitatory postsynaptic currents (mEPSC) was decreased, 3) neurofilament heavy (NFH) levels were reduced and no compensatory increases in other filament subunits were observed, and 4) the movement of mitochondria and to a lesser extent lysosomes was increased. Our findings elaborate the functional roles of NFL in human motor neurons. NFL is not only a structural protein forming neurofilaments and filling the axonal cytoplasm, but our study supports the role of NFL in the regulation of synaptic transmission and organelle trafficking. To rescue the NFL deficiency in the patient-specific nonsense mutant motor neurons, we used three drugs, amlexanox, ataluren (PTC-124), and gentamicin to induce translational read-through or inhibit nonsense-mediated decay. However, the drugs failed to increase the amount of NFL protein to detectable levels and were toxic to iPSC-derived motor neurons.

## Introduction

Neurofilaments are multimeric intermediate filaments in human neurons described to fill the axonal cytoplasm, establish caliber growth, and provide structural support (Kornreich et al., 2016; Yuan et al., 2017). They are 10 nm thick (Schmitt and Geren, 1950; Herrmann and Aebi, 2016) filaments formed by type IV intermediate filament subunits α-internexin (INA); neurofilament light (NFL), medium (NFM), and heavy (NFH); and peripherin (PRPH) (Ching and Liem, 1993; Lee et al., 1993; Yuan et al., 2017). The five neurofilament proteins co-assemble in different combinations depending on the neuron type and developmental stage (Kirkcaldie and Dwyer, 2017). Accumulations of neurofilaments are a pathological feature of several neurodegenerative diseases (Didonna and Opal, 2019), including amyotrophic lateral sclerosis (ALS), Alzheimer’s, and Parkinson’s diseases. Moreover, NFL as a highly abundant protein in neurons shows promise as a serum biomarker for neuronal disruption in various neurological diseases, such as ALS (Verde et al., 2021) and multiple sclerosis (Ferreira-Atuesta et al., 2021).

Mutations, dominant missense (Mersiyanova et al., 2000; Yoshihara et al., 2002; Jordanova et al., 2003; Stone et al., 2019), and recessive nonsense (Abe et al., 2009; Yum et al., 2009; Fu and Yuan, 2018; Sainio et al., 2018; Stone et al., 2019) in the *neurofilament light* (*NEFL*) gene cause Charcot–Marie–Tooth neuropathy (CMT), highlighting the important role of neurofilaments in motor and sensory neurons. To date, more than 25 dominant missense mutations causing CMT2E and six recessive nonsense mutations resulting in CMT1F are reported (Stone et al., 2021). CMT2E and CMT1F are both characterized as slowly progressive, length-dependent axonal neuropathies with highly variable disease onset. Symptoms include distal muscle weakness and atrophy. Patients with recessive CMT1F have reduced nerve conduction velocity and are, therefore, characterized as demyelinating even though myelin loss is not the primary cause of symptoms (Stone et al., 2021).

Effects of pathogenic missense mutations in NFL are studied in mouse and cell models. *Nefl* N98S knock-in mice displayed a tremor phenotype with hindlimb clasping (Adebola et al., 2015) as well as reduced nerve-conduction velocity (Lancaster et al., 2018). Furthermore, the mice showed neurofilament aggregates in the spinal cord and cerebellum as well as reduced size and amount of neurofilaments in myelinated axons (Adebola et al., 2015; Lancaster et al., 2018). Neurofilament accumulations and reduced number of neurofilaments were also observed in cultured dorsal root ganglia neurons of the mice (Zhao et al., 2017). In addition, stem cell–derived neuron models of dominant *NEFL* missense mutations show neurofilament aggregates and reduced mitochondrial transport (Saporta et al., 2015; Feliciano et al., 2021; Van Lent et al., 2021). Therefore, the *NEFL* missense mutations seem to cause neuropathy through accumulation of neurofilaments in the soma, which reduces their amount and functionality in axons.

Similarly to the missense mutations, recessive nonsense mutations in *NEFL* are suggested to lead to a toxic aggregation mechanism through formation of truncated NFL. However, we showed recently that a homozygous nonsense mutation in *NEFL* led to a complete absence of NFL instead of a truncated protein in patient-specific stem cell–derived neurons (Sainio et al., 2018). Contrary to nonsense mutations in humans causing early onset severe neuropathy, *Nefl* knockout (KO) mice displayed minor phenotypes, such as slower recovery from nerve crush injury (Zhu et al., 1997), disruption in neurite network (Liu et al., 2013), and reduction in locomotor activity (Dubois et al., 2005; Yuan et al., 2018). Cultured mouse motor neurons lacking NFL show reduction in dendritic arborization (Zhang et al., 2002) and increased mitochondrial motility (Perrot and Julien, 2009).

To investigate the detailed functional effects of NFL loss in human motor neurons, we here use stem cell–derived motor neurons and compare the patient-specific nonsense mutant and genome edited *NEFL* KO to isogenic control neurons. We uncovered significant alterations in axonal caliber, organelle trafficking, and electrophysiological properties in both nonsense mutant and *NEFL* KO motor neurons. Our results suggest that, in human motor neurons, NFL is critical for axon caliber and is also required for organelle trafficking and synaptic transmission.

## Results

### *NEFL* KO iPSC Differentiate into Motor Neurons

**FIGURE 1:**
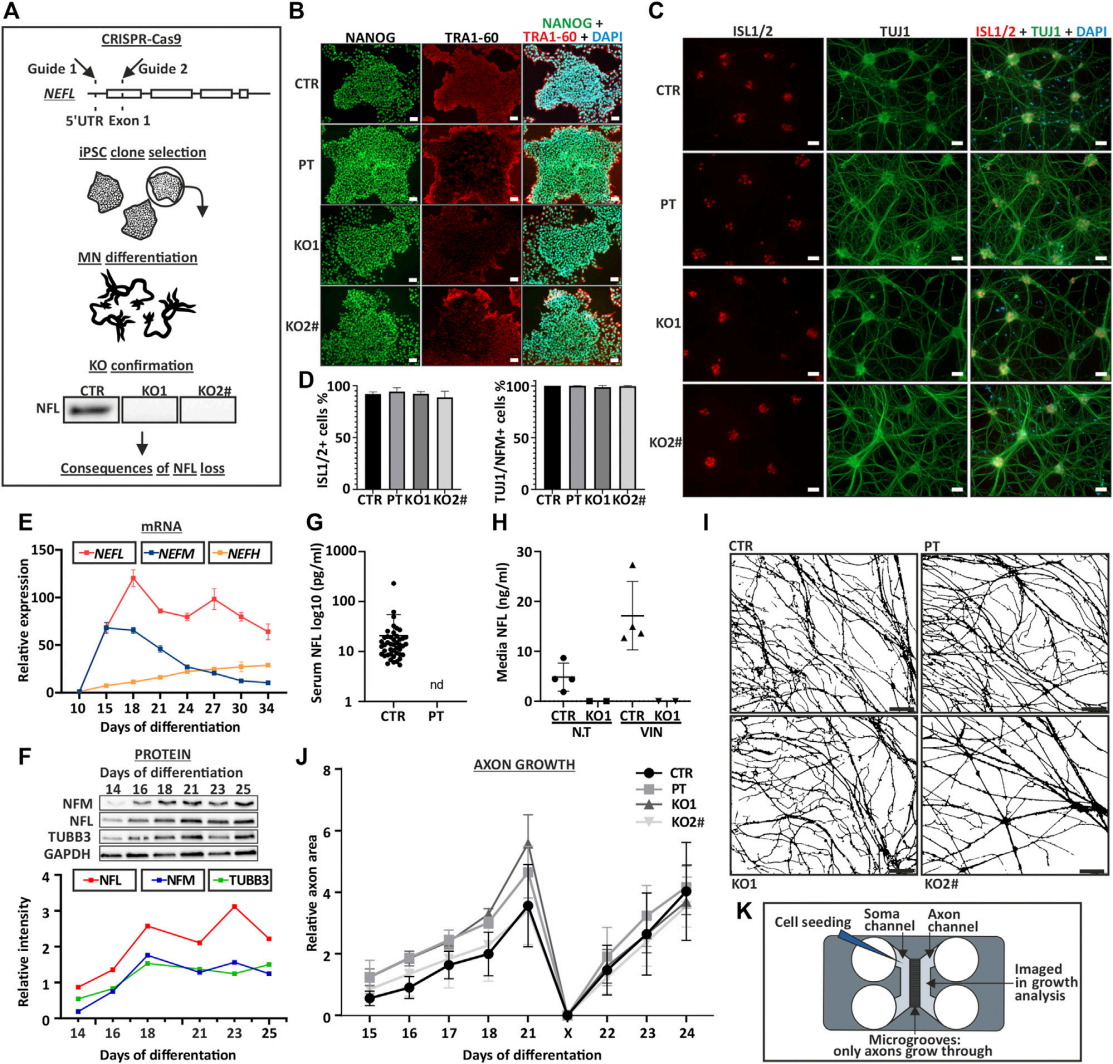
Characterization of *NEFL* KO iPSC and motor neurons. **(A)** *NEFL* was knocked out from control iPSC with two guides targeting 5′UTR and Exon 1. iPSC clones were selected for differentiation. KO candidates were differentiated into motor neurons and absence of full-length NFL was confirmed by immunoblotting. Cell lines with no signal in immunoblotting were used for further assays. **(B)** *NEFL* KO and patient (PT) iPSC lines are pluripotent. Control (CTR), *NEFL* KO (KO1 and KO2) and PT iPSC express pluripotency markers NANOG (green) and TRA-1-60 (red), analyzed by immunocytochemistry (nuclei counterstain with DAPI, blue). Scale bar 50 µm. **(C)** KO and PT iPSC differentiate with similar efficiency as the isogenic control. Differentiation efficiency of Day 35 motor neurons was assayed by counting ISL1/2 (red) positive and TUJ1 (green) or NFM (not shown) positive cells in immunocytochemistry. DAPI counterstain (blue). Scale bar 50 µm. **(D)** Quantification of C. Average % of positive nuclei/cytoplasm with standard deviation (SD). Six replicates from three independent differentiations with 18 frames. One-way ANOVA followed with Dunnett’s multiple comparison post hoc test vs. CTR. *p* > .05. **(E)** *NEFL*, *NEFM,* and *NEFH* mRNA expression during differentiation. *N* = 2-3 per time-point. Normalized to *ACTB* expression and to day 10 expression of respective gene. **(F)** NFL, NFM, and TUBB3 protein levels during differentiation. Normalized to GAPDH signal intensity. *N* = 1 per time-point. **(G)** NFL measurement by Simoa from serum of control individuals and PT. Each dot indicates an individual sample [control values are the same as in (Järvilehto et al., 2021)]. Nd = not detectable. Average with SD (pg/ml). **(H)** NFL measurement by Simoa from iPSC-MN culture media. Treatment with the neurotoxic drug vincristine (VIN), which induces axonal degeneration was used as a positive control. N.T = not treated. CTR *n* = 4 and KO1 *n* = 2. Average with SD (ng/ml). **(I)** Axonal growth is not affected by NFL loss. Masks of phase contrast images of day 21 motor neuron axons in a microfluidic device (Xona, 450 μm). Scale bar 100 µm. **(J)** Quantification of image I. axon area on axonal side of microfluidic device. Axonal growth and regrowth after mechanical axotomy is not affected by NFL loss. Average relative area of axonal coverage and SEM. *n* = 3 independent experiments/cell line with 5 frames analyzed per time point. Growth normalized to average day 15 axon area per experiment. X indicates axotomy. Two-way ANOVA followed with Tukey’s multiple comparison post hoc test *p* > .05. **(** **K** **)** Schematic of a microfluidic device used for axon analysis. ^#^ indicates abnormal karyotype.

Here, we studied the previously generated patient-specific induced pluripotent stem cells (iPSC) carrying the homozygous *NEFL* nonsense mutation p. Arg367* (PT) (Sainio et al., 2018) as well as used genome editing to KO *NEFL* in the 46.11 control iPSC line (CTR) (Trokovic et al., 2015). For the editing, we used CRISPR-Cas9 with two guides targeting the 5′UTR and exon 1 of *NEFL* (Figure 1A). Edited isogenic *NEFL* KO iPSC lines (KO1 and KO2) were confirmed to be pluripotent by positivity for NANOG and TRA-1-60 in immunocytochemistry (Figure 1B). All iPSC lines represented a normal karyotype except KO2 that had a deletion in the long arm of chromosome 18 (46, XY, del(18)(q12q22)) (Supplementary Figure S1). As we had included the KO2 iPSC line in several experiments, its results are shown for comparison despite the chromosomal alteration.

In comparison to the differentiation efficiency in our previous study (Sainio et al., 2018), the use of an optimized differentiation protocol here (Maury et al., 2015; Guo et al., 2017) resulted in a higher motor neuron purity. All iPSC lines (CTR, PT, KO1, KO2) differentiated into motor neurons similarly. On day 35 of differentiation, more than 90% of postmitotic DAPI stained cells were Islet-1/2 (ISL1/2) positive, and more than 98% were positive for NFM or B-tubulin III (TUBB3, antibody clone TUJ1) (Figures 1C,D). In this differentiation protocol, *NEFL* expression initiates after day 10 and peaks after day 15 (Figure 1E). NFL and NFM protein levels increase until day 18 after which they are stabilized (Figure 1F).

**FIGURE 2:**
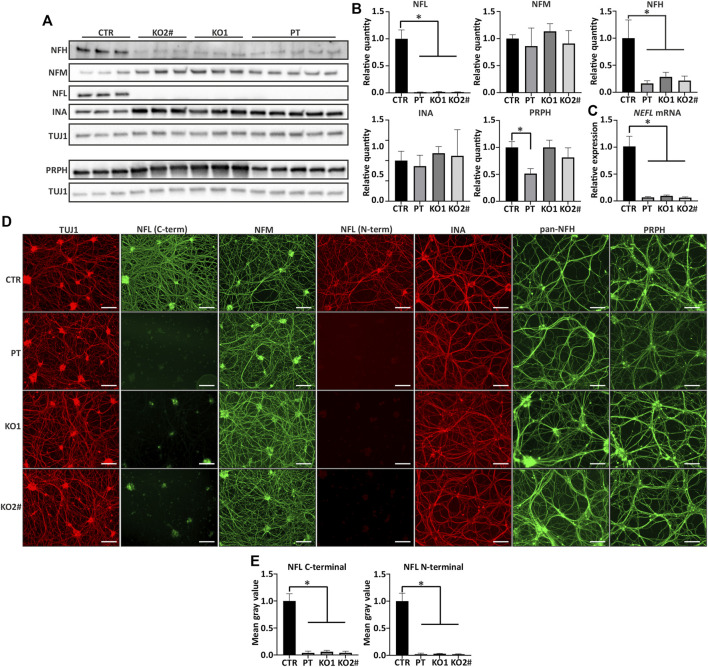
Other filament proteins are expressed in *NEFL* KO and patient iPSC-MN. **(A)** *NEFL* KO motor neurons express other neurofilament proteins. Representative immunoblot showing presence of all other tested neurofilament proteins in *NELF* KO and PT Day 35 motor neurons. NFL = NFL antibody (Sigma) and TUJ1 = TUBB3 antibody. **(B)** Quantification of A. Full-length NFL is indistinguishable from background (*p* < .0001) and NFH is reduced in KO and patient motor neurons (*p* < .0001). NFM or INA levels are not changed, and PRPH is reduced only in PT (*p* < .0001). *n* = 6-7 from two independent differentiations. Signal intensity normalized to TUBB3 protein signal. **(C)** RT-qPCR shows reduction (*p* < .0001) in *NEFL* mRNA expression. The residual amount of *NEFL* mRNA is approximately 10% of control levels. Expression levels are normalized to *TUBB3*. *n* = 6-7 from two independent differentiations. **(D)** All differentiated motor neurons (days 35–39) grow elaborate neurite networks positive for TUBB3 (TUJ1), NFM, INA, NFH, and PRPH. Scale bar 100 µm. **(E)** Quantification of NFL from immunocytochemistry shows reduction in NFL signal with C- and N-terminal antibodies (*p* < .0001). *n* = 4 from two independent differentiations. Data shown in all as mean with SD. *p < .0001. One-way ANOVA followed with Dunnett’s multiple comparison post hoc test vs. CTR. ^#^ indicates abnormal karyotype.

We confirmed that the *NEFL* KO and PT iPSC-derived motor neurons (iPSC-MN) lacked full-length NFL by immunoblotting (Figures 1A, 2A and Supplementary Figure S2) and immunocytochemistry (Figures 2D,E). Furthermore, NFL was absent in patient serum and in the culture media of iPSC-MN of KO1 as measured by single-molecule array (Simoa) (Figures 1G,H).

### Axonal Growth is not Affected by NFL Loss in Human MN

The establishment of the MN neurite network was not affected by the loss of NFL as seen by immunocytochemistry (Figures 1C, 2D). We investigated the axon growth of iPSC-MN using microfluidic devices, which contain microgrooves physically separating neuronal somas and dendrites from axons. We analyzed the axon-specific growth by area covered on the axonal compartment of the microfluidic device (Nijssen et al., 2018; Klim et al., 2019).

iPSC-MN axons grew in length and branched with similar speed, covering the same area, in neurons lacking NFL compared with isogenic control neurons (Supplementary Figure S3). In addition, axonal regrowth after mechanical axotomy (the removal of axons from axonal compartment with forceful aspiration) was not reduced in iPSC-MN without NFL (Figures 1I–K). We also analyzed axonal branching in the microfluidic system with Sholl-analysis (number of axonal crossings per section), which revealed no differences between the cell lines (Supplementary Figure S4).

### Alterations in Neurofilament Composition in Absence of NFL

We assessed the filament protein levels to elucidate the composition of neuronal cytoskeleton in the absence of NFL. Apart from NFL, all other investigated intermediate filaments, which are involved in neurofilament assembly in mature neurons, were detectable by immunoblotting (Figure 2A) and immunocytochemistry (Figure 2D). The protein levels of NFM and INA were unchanged while NFH levels were significantly lower in iPSC-MN lacking NFL (one-way ANOVA, multiple comparisons vs. CTR *p* < .0001) (Figure 2B). Furthermore, *NEFH* mRNA was reduced to approximately 65% in cell lines lacking NFL while *NEFM* levels were unchanged (Supplementary Figure S5). By immunocytochemistry, filament protein distribution was not different between iPSC-MN lines, indicating their incorporation into the filament network in the absence of NFL (Figure 2D). Quantification of reduced NFL signal in immunocytochemistry with N- and C-terminal antibodies is shown in Figure 2E (one-way ANOVA, multiple comparisons vs. CTR *p* < .0001).

Next, we investigated NFL oligomers by long immunoblot exposure to detect high molecular weight bands. These oligomers represent neurofilament subunits containing homo- and heteropolymers of NFL. High molecular weight (>68 kDa) oligomers reactive to NFL antibodies were observed in control but not in iPSC-MN lacking NFL (Supplementary Figure S6). Signal from NFM, PRPH, and INA oligomers was detected in absence of NFL, indicating their presence as neurofilament building blocks devoid of NFL (Supplementary Figure S6). In addition, whereas clear bands were visualized in control iPSC-MN, no NFL was detected in non-RIPA (Triton) soluble fractions of PT and KO iPSC-MN (Supplementary Figure S7).

**FIGURE 3:**
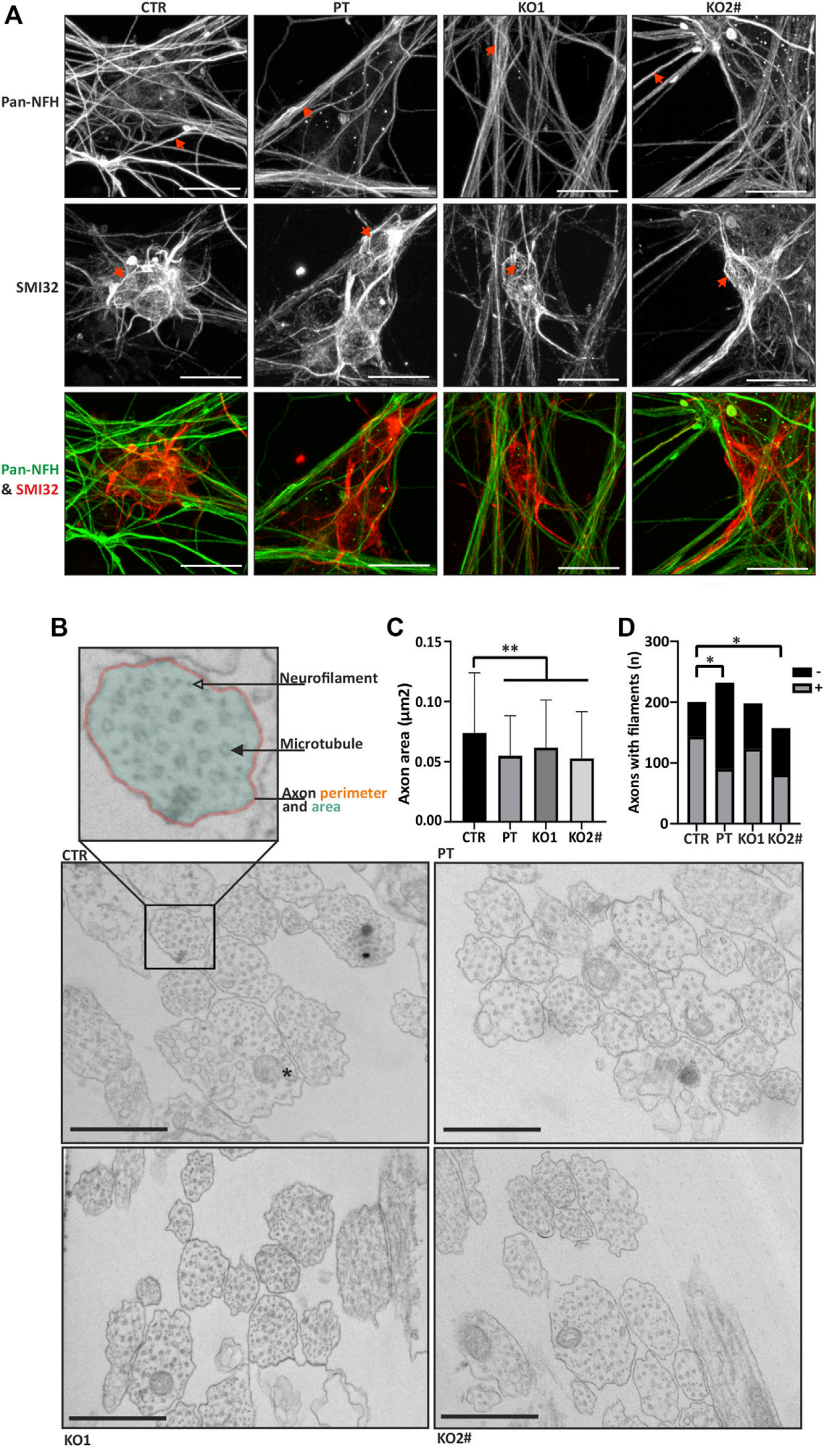
iPSC-MN lacking NFL form filamentous structures and have a reduced axonal caliber. **(A)** Motor neurons (day 35) in all cell lines have filamentous structures in the neurites and soma. Spinning disc confocal z-stack imaging with maximum intensity projections reveals neurofilament-like structure in motor neurons. SMI32 red and pan-NFH (green). Scale bar 20 µm. Arrows indicate filamentous structures in iPSC-MNs. **(B)** Transmission electron microscopy reveals differences in axonal structure. Axon perimeter (orange outline), area (transparent teal fill) and presence of microtubules (filled arrowhead), filaments (transparent arrowhead) and mitochondria (asterisk in CTR panel) were assayed. Scale bar 500 nm. **(C)** Quantification of electron micrographs in B. confirms decreased axon area (PT/KO1/KO2 vs. CTR *p* < .0004) in patient and knockout iPSC-MNs. *n* = 157–232 axons per cell line. Data shown as mean with SD. **p < .001. One-way ANOVA followed with Dunnett’s multiple comparison post-hoc test vs. CTR. **(D)** Proportion of neurofilament positive axons is reduced in PT (*p < .05, Fishers exact) and KO2 (*p < .05, Fishers exact). ^#^ indicates abnormal karyotype.

Furthermore, we studied the neurofilament oligomeric structures with z-stack confocal microscopy and maximum intensity projections. Imaging of NFH signal revealed filamentous structures within the neuron cytoplasm and neurites in iPSC-MN lacking NFL (Figure 3A). Cytoplasmic neurofilaments were visualized with SMI32 antibody against nonphosphorylated NFH and neurite filaments with a pan-NFH antibody. In contrast to WB, no difference in NFH signal intensity was detectable between control and KOs (data not shown). In conclusion, our immunoblotting and immunocytochemical analysis showed that human iPSC-MN assembled oligomeric neurofilaments with altered composition in the absence of NFL *in vitro*.

### Axon Caliber is Reduced in iPSC-MN in the Absence of NFL

Next, we investigated the axonal structure with transmission electron microscopy (TEM). Fixed iPSC-MN axons were cross-sectioned, imaged with TEM and axonal shape characteristics were analyzed. Axon area was reduced in the iPSC-MN axons lacking NFL in comparison to controls (Figures 3B,C). Average (±SD) axon area of neurons without NFL was 0.062 μm^2^ (±0.040) in KO1, 0.053 μm^2^ (±0.039) in KO2 and 0.055 μm^2^ (±0.033) in PT, whereas the control axon area was significantly greater with an average of 0.074 μm^2^ (±0.050) (one-way ANOVA multiple comparisons vs. CTR *p* < .0004). Presence of neurofilaments was analyzed by categorizing microtubule containing axons into neurofilament bundle positive and negative axons. PT (39%) and KO2 (52%) neurons had less neurofilament positive axons than control (72%) (*p* < .05, Fisher’s exact), whereas KO1 did not reach statistical significance with 62% of axons containing neurofilaments (Figure 3D). These structural changes in axons guided us to investigate the functional effects of NFL loss.

### Absence of NFL Leads to Synaptic Defects in Patient-Derived and Isogenic KO iPSC-MN

**FIGURE 4:**
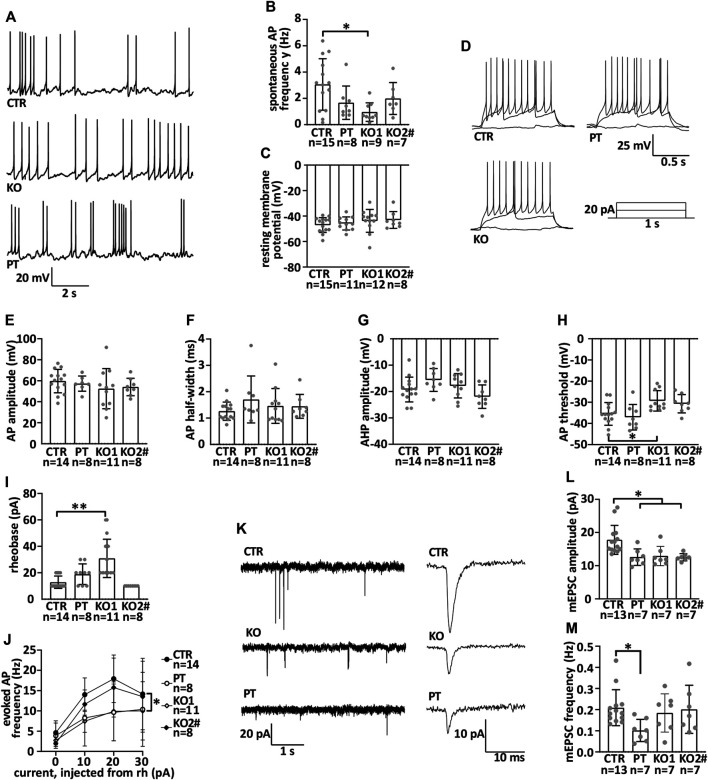
iPSC-MN lacking NFL differentiate into functional motor neurons with reduced miniature excitatory postsynaptic current amplitude. Motor neuron electrophysiological characteristics were measured by whole cell patch-clamp during the seventh week of differentiation. **(A)** Example traces of spontaneous action potentials. **(B)** Frequency of spontaneous action potentials in current-clamp (CTR vs. KO1 multiple-comparison *p* = .0106). **(C)** Resting membrane potential. **(D)** Example traces of action potentials evoked by depolarization and a scheme of the depolarizing current injected. **(E)** Action potential amplitude. **(F)** Action potential half-width. **(G)** After-hyperpolarizing potential amplitude. **(H)** Action potential threshold (CTR vs. KO1 *p* = .0322). **(I)** Rheobase (CTR vs. KO1 *p* < .0001). **(J)**. Evoked action potential frequency (*p* = .02, two-way repeated measures ANOVA). **(K)** Example traces of miniature excitatory post synaptic currents (mEPSCs) and averaged mEPSCs shown with the expanded time scale. **(L)**. Amplitude of mEPSC is reduced in iPSC-MNs lacking NFL (PT/KO1/KO2 vs. CTR *p* < .0170). **(M)** Frequency of mEPSC in iPSC-MNs. Data shown as individual measurements/neurons and mean with SD. *p < .05, **p < .0001. One-way ANOVA followed with Dunnett’s multiple comparison post hoc test vs. CTR and two-way ANOVA followed with Tukey’s multiple comparison post hoc test for J. ^#^ indicates abnormal karyotype.

We then analyzed the electrophysiological functionality of iPSC-MN by whole-cell patch-clamp to assess the effect of NFL loss. All iPSC-MN lines displayed spontaneous action potentials in current-clamp mode (Figure 4A). The amplitude (not shown) and frequency (Figure 4B) of the action potentials did not differ between neuron lines. Thus, no consistent differences were seen in spontaneous action potential characteristics between CTR and NFL KO or PT iPSC-MN. The neurons retained an evenly negative resting membrane potential (Figure 4C). Additionally, all cell lines fired evoked action potentials with similar properties (Figure 4D). There was no difference in action potential amplitude (Figure 4E), half-width (Figure 4F), or after-hyperpolarization potential amplitude (AHP) (Figure 4G). Only isolated differences were seen in action potential (AP) threshold (one-way ANOVA, CTR vs. KO1 multiple-comparison *p* = .0322) (Figure 4H), rheobase (one-way ANOVA, CTR vs. KO1 multiple-comparison *p* < .0001) (Figure 4I), and evoked AP frequency (reduced in KO1 and PT, *p* = .02, two-way ANOVA) (Figure 4J), but these were not consistent across lines. The capacitance and input resistance of the motor neurons were similar (data not shown). In conclusion, the iPSC-MN lacking NFL differentiated into functional neurons with similar electrophysiological characteristics as controls.

NFL is shown to interact with glutamatergic receptors in mice (Ehlers et al., 1998; Yuan et al., 2015; Yuan and Nixon, 2016; Yuan et al., 2018). To study the function of glutamatergic synapses, we recorded AMPA receptor-mediated miniature excitatory postsynaptic currents (mEPSCs) in a whole-cell patch-clamp configuration in our neuronal cultures [for details, see (Harjuhaahto et al., 2020)]. AMPA mEPSCs are generally used to investigate the function of glutamatergic synapses [see, e.g., (Huupponen et al., 2013)] (Figure 4K). NFL loss reduced the amplitude of mEPSCs (Figure 4L) (one-way ANOVA, multiple comparisons vs. CTR *p* < .0170). The frequency of events was unaltered (Figure 4M). Therefore, NFL loss resulted in a specific and robust reduction in mEPSC amplitude indicating alterations in postsynaptic glutamatergic receptor functionality or neurotransmitter quantal size in the human motor neurons lacking NFL.

### Axonal Transport of Mitochondria is Increased in Absence of NFL

**FIGURE 5:**
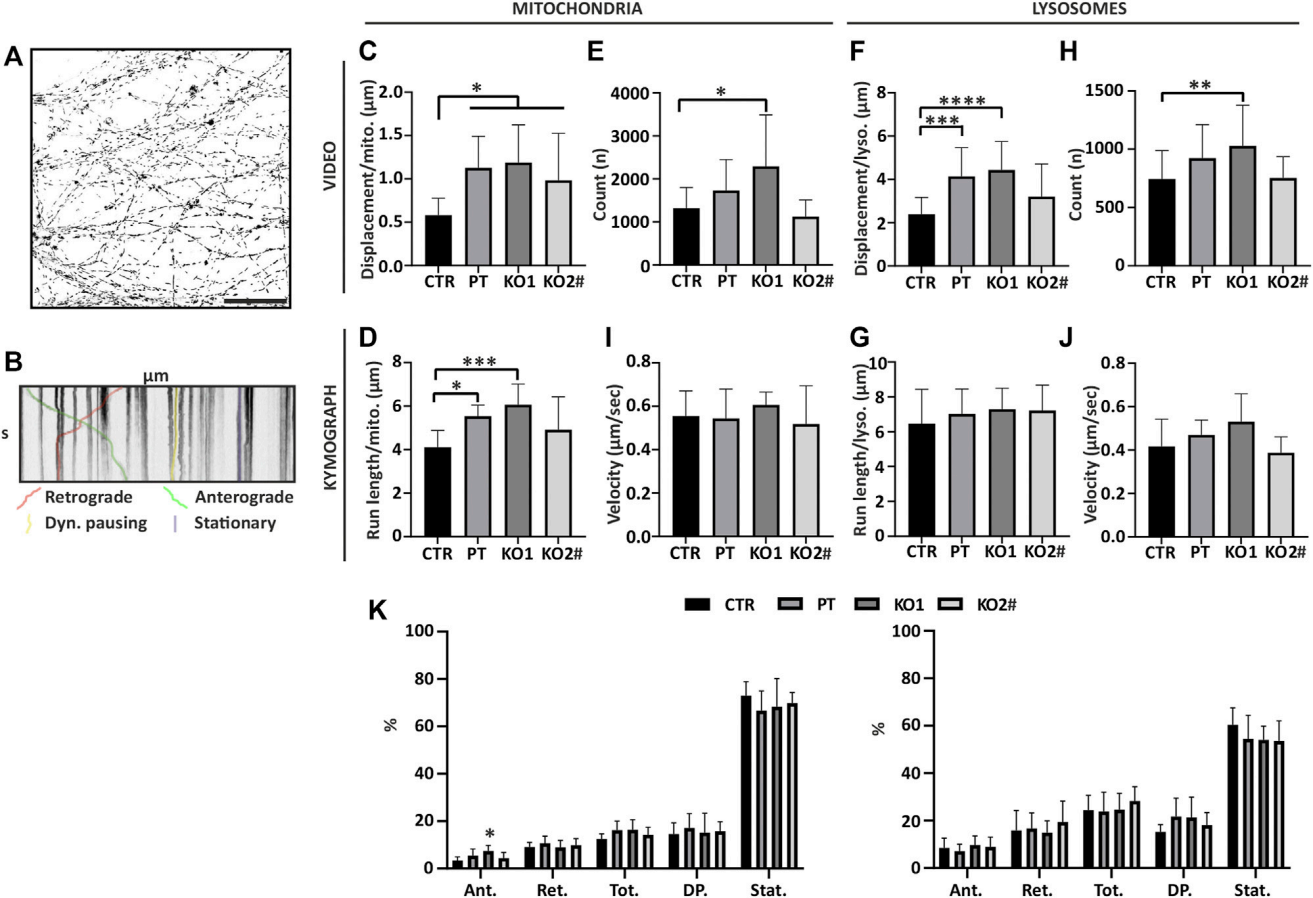
Movement of mitochondria and lysosomes is increased in iPSC-MN axons lacking NFL. Mitochondria and lysosome movement along the axons of motor neurons reveals increased movement in PT and KO cells. **(A)** Mask of CTR mitochondria tracking video frame 1. Total axon mitochondrial movements by time-lapse imaging of entire micrographic fields (*n* = 15 fields from three independent differentiations). Scale bar 50 µm. **(B)** Organelle movement by kymographs of axonal segments (*n* = 9 micrograph frames from three independent differentiations, total 45 axon segments/cell line). Shown is an example kymograph with retrograde and anterograde movement, stationary mitochondria, and mitochondria with less than 0.2 µm movement during 60 s (Dyn. Pausing = Dynamic pausing). **(C)** Mitochondria total displacement/number of mitochondria is increased in axons lacking NFL (PT/KO1/KO2 vs. CTR *p* > .0173). **(D)** Average run length of a mitochondria normalized to the count of organelles per kymograph is increased in PT and KO1 (CTR vs. KO1 *p* = .0007, CTR vs. PT *p* = .0138). **(E)** Number of mitochondria per video (first frame) (CTR vs. KO1 *p* = .0013). **(F)** Lysosome total displacement/number of lysosomes is increased in PT and KO1 motor neuron axons (CTR vs. KO1 *p* < .0001 and CTR vs. PT *p* = .0003). **(G)** Average run length of a lysosome normalized to the count of lysosomes. **(H)** Number of lysosomes per video (first frame) (CTR vs. KO1 *p* = .0099). **(I)** Average velocity of a moving mitochondria. **(J)**. Average velocity of a moving lysosomes. **(K)** Proportions of anterograde moving (Ant.), retrograde moving (Ret.), anterograde and retrograde combined (Tot.), dynamic pausing (DP.) and stationary (Stat.) organelles in kymographs. KO1 iPSC-MN have proportionally more anterograde moving mitochondria (CTR vs. KO1 *p* = .0029). Data shown as mean with SD. *p < .05, **p <.01, ***p < 0.001, ****p < .0001. One-way ANOVA followed with Dunnett’s multiple comparison post hoc test vs. CTR. ^#^ indicates abnormal karyotype.

Reduced organelle trafficking is implicated in multiple neurodegenerative diseases (Guo et al., 2020; Fazal et al., 2021) and in selective length-dependent degeneration of the longest axons as well as with dominant *NEFL* missense mutations (Gentil et al., 2012; Saporta et al., 2015; Perez-Siles et al., 2020; Van Lent et al., 2021). To address whether organelle movement is affected in motor neurons lacking NFL, we assayed mitochondrial and lysosome trafficking in live axons in microfluidic devices with organelle specific live-cell dyes, Mito- and Lysotracker (Figure 5).

We analyzed the imaged data as videos of hundreds of moving organelles per movie (Figure 5A) to assay the robust changes of total organelle movement. We also selected individual axons with clear trackable organelles (Figure 5B) to analyze the detailed characteristics of organelle movement.

Organelles were evenly distributed along the axon length in all cell lines (data not shown). Overall mitochondrial movement, anterograde and retrograde combined, was increased in motor neuron axons of NFL KO and PT as assayed by the video analysis (Figure 5C) (one-way ANOVA multiple comparison vs. CTR *p* > .0173) as well as by analyzing individual axon kymographs (Figure 5D) (one-way ANOVA, multiple comparisons CTR vs. KO1 *p* = .0007, CTR vs. PT *p* = .0138, CTR vs. KO2 *p* > .05). An even number of mitochondria were detected in imaged video frames except that KO1 had, on average, more axons and mitochondria per frame (one-way ANOVA, multiple comparison CTR vs. KO1 *p* = .0013) (Figure 5E). Lysosome movement was increased in PT and KO1 motor neurons in video analysis (Figure 5F) (one-way ANOVA, multiple comparison CTR vs. KO1 *p* < .0001 and CTR vs. PT *p* = .0003), but not in analysis of individual axons (Figure 5G). Therefore, the trafficking of mitochondria and, to a lesser extent, lysosomes was increased in the axons devoid of NFL, normalized to the number of detected organelles. As with mitochondria, lysosomes were evenly distributed along the axons in all cell lines in video analysis except KO1, which had more detectable organelles per video than other cell lines (one-way ANOVA, multiple comparison CTR vs. KO1 *p* = 0.0099) (Figure 5H). We used the individual axon kymographs to assay the speed, directionality, and proportions of moving organelles. The velocity of organelles was not altered in motor neurons lacking NFL (Figures 5I,J). However, the proportion of anterograde moving mitochondria was increased in KO1 (Figure 5K) (one-way ANOVA, multiple comparison CTR vs. KO1 *p* = .0029). The proportions of moving lysosomes were similar in all cell lines. Moreover, directional run length and velocity analysis of organelles revealed no consistent differences (data not shown). In contrast to *NEFL* missense mutations, the overall movement, anterograde and retrograde combined, of mitochondria was increased in the absence of NFL. The proportion of transported mitochondria or their speed was unaltered, but the moving mitochondria tended to move longer distances between pauses.

### Nonsense Suppression did not Increase the Production of Full-Length NFL in Patient-Derived Motor Neurons

We previously showed that, in patient-derived neurons, the nonsense mutation containing *NEFL* mRNA is degraded through nonsense mediated decay (NMD) (Sainio et al., 2018) in which the premature stop-codon is detected by ribosome (Nagel-Wolfrum et al., 2016). Nevertheless, some *NEFL* mRNA was retained in the PT neurons. Similarly, here, in KO iPSC-MN, approximately 10% of *NEFL* mRNA could be detected by qPCR using primers located in the 3′ end of the mRNA (one-way ANOVA, multiple comparisons vs. CTR *p* < .0001) (Figure 2C). These residual transcripts lacked the region that was targeted by guideRNAs containing the beginning of exon 1. This persistence of a low level of *NEFL* mRNA is likely to result from *NEFL* being among the most highly transcribed genes in human neurons (Sainio et al., 2018).

Occasionally, a premature stop-codon is not recognized by the NMD machinery, and an amino acid is incorporated instead of translational stop. Leakage of the ribosome can be increased with translational readthrough inducing drugs (TRIDs), or NMD can be inhibited with NMD inhibitors (NMDi) to increase the amount of nonsense mutation containing mRNA (Malik et al., 2010; Banning et al., 2018). TRIDs are, for example, shown to increase the expression of full-length COL7A1 in keratinocytes and fibroblasts in epidermolysis bullosa (Atanasova et al., 2017), but to our knowledge, TRIDs have not been tested in iPSC-derived neuronal models.

**FIGURE 6:**
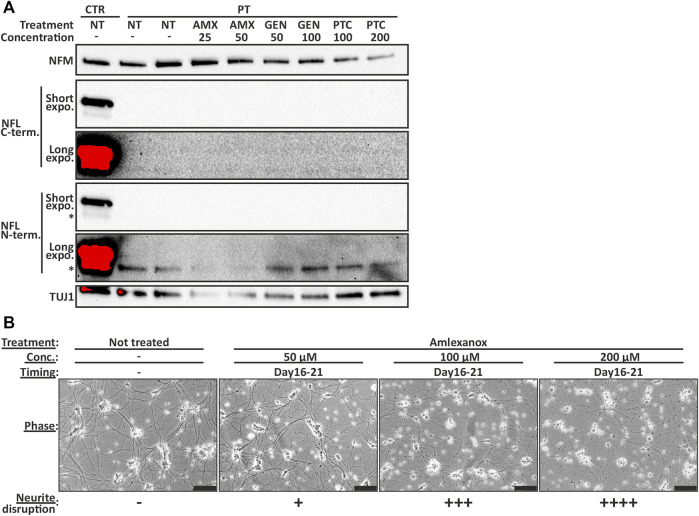
NFL protein is not induced to detectable levels by TRID or NMDi treatment in PT iPSC-MN. **(A)** Immunoblotting series (same blot sequentially analyzed with different antibodies) shows no detectable full-length NFL in treated patient motor neurons. Short and long exposure times shown with two separate NFL antibodies. Treatment between days 16–25 with 25 and 50 µM of amlexanox (AMX), 50 and 100 μg/ml of gentamicin (GEN) and 100 and 200 µM of PTC-124 (PTC). Asterisk indicates an unspecific band detected by the NFL antibody. Concentration as µM for AMX and PTC, and µg/ml for GEN. **(B)** Representative images of AMX toxicity in day 21 motor neurons (treated for 5 days). Motor neuron health is categorized to—no effect, + some neurite swellings, ++ swellings in most neurites, +++ neurite disruption and ++++ severe neurite disruption and cell death. Higher concentration causes more severe disruption. Scale bar 100 µm.

We investigated the ability of TRIDs ataluren (PTC-124) and gentamicin (GEN) and NMDi amlexanox (AMX) to increase the endogenous full-length NFL protein amount in PT iPSC-MN. The treatment was initiated on day 14 or 16 of differentiation and continued for 7–12 days. However, the full-length NFL protein amount was not increased to detectable levels in PT iPSC-MN with any drug, concentration, or treatment time tested in this study. In addition, no truncated protein was detectable (Figure 6A). Furthermore, NMDi and TRIDs were toxic to the developing motor neurons causing neurite disruption and ultimately cell death (Figure 6B). The lowest concentrations causing neurite disruption were 25 µM of AMX on days 16–25, 200 µM of PTC-124 on days 16–25, and 125 μg/ml of GEN on days 14–26 of differentiation. Therefore, PTC-124 treatment was the most tolerated resulting in neurite disruption only with the highest concentrations. AMX and GEN toxicity was increased by drug concentration and earlier administration. In conclusion, the tested TRIDs or NMDi were not able to rescue NFL loss in patient neurons.

## Discussion

NFL has emerged as an important filament protein in neurodegenerative diseases. We recently showed that early onset CMT can result from absence of NFL. Here, we characterize in detail the functional effects of NFL loss in human iPSC-MN. We utilized an efficient differentiation protocol to produce high purity MN cultures and microfluidics to assay axon-specific properties and used genome edited isogenic iPSC lines for optimal comparisons. With these human neuronal models, we could detect robust effects of NFL loss. We show significant changes in electrophysiological function, organelle trafficking, and axon structure in human iPSC-MN lacking NFL.

Developing mouse motor neurons are shown to express high amounts of NFL (Pachter and Liem, 1984; Faussone-Pellegrini et al., 1999; Kirkcaldie and Dwyer, 2017), and similarly, in the differentiation protocol used by us here, NFL expression is induced from early neuronal progenitor stages, suggesting that it is important for the growth of MN and axons. However, using two different iPSC-MN differentiation protocols [(Sainio et al., 2018), and here] we have now shown that NFL is dispensable for differentiation and axon growth of human MN in culture, and neurofilaments can be detected in NFL-null axons. Compensation by other intermediate filaments could, thus, be expected but was not observed. Instead, the NFH amount was reduced. Similar findings are reported in studies of *Nefl* KO mice, which show the presence of neurofilaments *in vivo* (Zhu et al., 1997; Yuan et al., 2003; Liu et al., 2013; Adebola et al., 2015; Yuan et al., 2018) with decreased NFH and no compensation by NFM or INA (Zhu et al., 1997; Yuan et al., 2003). Neurofilaments are shown to be responsible for axonal caliber growth in mammals (Ohara et al., 1993; Zhu et al., 1997; Jacomy et al., 1999; Rao et al., 2012; Yuan et al., 2017), and the axonal caliber was also reduced in our iPSC-MN lacking NFL. Thus, the human iPSC-MN model recapitulates the effects of NFL loss on neurofilament composition and axon structure that have been found in mice *in vivo*.

Dynamic regulation of mitochondrial form and transport is critical in long peripheral axons, and its defects are one of the main pathways affected in CMT (Markworth et al., 2021). NFL is shown to interact with Myosin Va (MyoVa) and, therefore, function as an anchor through the MyoVa interaction for mitochondria and other organelles (Rao et al., 2002; Rao et al., 2011). Thus, neurofilaments regulate organelle distribution and trafficking in mammalian neurons and axons (Kim et al., 2002; Wagner et al., 2003; Balastik et al., 2008; Yadav et al., 2016), and missense mutations leading to NFL aggregates cause reduction in organelle trafficking. The aggregated NFL can possibly sequester proteins needed for transport in the neuron, reducing organelle movement in CMT neurites (Gentil et al., 2012; Saporta et al., 2015). We show that the loss of NFL in the soma and axons results in increased movement of mitochondria and to a lesser extent lysosomes, possibly caused by reduced interactions between the cytoskeleton and moving cargo (Wagner et al., 2003; Gentil et al., 2012). Similarly, more persistent trafficking of mitochondria and lysosomes are seen in mouse *Nefl* KO primary dorsal root ganglion neurons (Perrot and Julien, 2009). Thus, our results support the idea that neurofilaments can serve as a docking site for these organelles through interaction with MyoVa, affecting the frequency of their pausing and, thus, slowing down their axonal transport and that NFL, in particular, has a role in this regulation (Rao et al., 2002; Wagner et al., 2003; Perrot and Julien, 2009). Mitochondria contribute to energy metabolism and calcium buffering along the axon and in synapses (Smith and Gallo, 2018), and thus, hypothetically, the reduction in the interaction with the axonal cytoskeleton could lead to reduced number of stationary synaptic and calcium buffering mitochondria and to local energy and metabolic deficiencies.

Our detailed electrophysiological analysis revealed that the iPSC-MN fired spontaneous and evoked action potentials with and without NFL. However, a reduction in the amplitude of mEPSCs but not in their frequency was found in the absence of NFL. The robust reduction in mEPSCs amplitude can be a consequence of reduced number of receptors in the postsynaptic cell or reduced release of neurotransmitter per synaptic vesicle. In addition to neurofilaments regulating the electrophysiological parameters via axon caliber (Križ et al., 2000), NFL is shown to associate with glutamatergic receptors, possibly functioning as an anchor and reducing receptor degradation (Ehlers et al., 1998; Yuan and Nixon, 2016; Bragina and Conti, 2018). *Nefl* KO is shown to reduce the number of glutamatergic receptors on the postsynaptic cortical murine neurons (Yuan et al., 2018). Moreover, NFM is shown to interact with dopaminergic receptors (Kim et al., 2002) and increase the number of the receptors on the postsynaptic membrane of neurons (Yuan et al., 2015). Further work on human models is required to confirm the interplay of receptors on the postsynaptic cell and neurofilaments. Long-term cultures of motor neurons lacking NFL could reveal impaired electrophysiological properties that were not seen in our study. Alternatively, we cannot exclude the possibility of abnormal neurofilament structure affecting synaptic vesicle trafficking or packing (Rao et al., 2011).

Nonsense suppression is an intriguing avenue of research with the aim to cure diseases caused by nonsense mutations (Nagel-Wolfrum et al., 2016). TRIDs have been tested in treatment trials for multiple disorders, such as cystic fibrosis (Wilschanski et al., 2003), Duchenne muscular dystrophy (Malik et al., 2010), and epidermolysis bullosa (Atanasova et al., 2017). Even though evidence of read-through mechanisms in different genes and mutations has aggregated during the years, it is still somewhat unclear why in certain situations read-through is possible and in some not. In an epidermolysis bullosa study, the nucleotide cytosine after the stop codon had the highest efficacy to be read through, but only when there is detectable basal read-through (Atanasova et al., 2017). Thus, in the case of our nonsense mutant patient-derived cells, which show no detectable basal read-through and the nucleotide after the stop codon is thymine, drug-induced read-through was unlikely and was not observed with any of the tested drugs. On the other hand, residual mRNA and the UGA stop codon in the patient would indicate some mRNA readthrough (Martins-Dias and Romão, 2021). Although only 10% of nonsense mutant *NEFL* mRNA is retained in patient-specific MN, it is a relatively large amount of mRNA because of the high overall expression of *NEFL*. However, strict mechanisms appear to prevent read-through of nonsense *NEFL* mRNA in MN. In addition, the tested TRIDs caused neurite disruption and neuronal death, which is in line with off-target TRID toxicity with lysosomal membranes (Laurent et al., 1982) and NMD defects causing neuronal pathology in humans (Alrahbeni et al., 2015). Nevertheless, the iPSC-MN with the *NEFL* nonsense mutation offer an interesting model to test the efficiency and toxicity of other nonsense suppressors as NFL is such an abundant protein and easily detected in MN and culture media.

A common pathological mechanism of axonal neurofilament dysregulation is likely to be responsible for neuropathy in the case of both dominant and recessive *NEFL* mutations as well as in other disorders caused by NFL aggregation (Zhang et al., 1997; Bomont et al., 2000; Evgrafov et al., 2004; Balastik et al., 2008; Didonna and Opal, 2019). In recessive nonsense mutations, the neurons are missing an integral protein of the neurofilament leading to neurofilament stoichiometry dysregulation in the axon and to alterations described in this study. In the case of aggregating mutant NFL and in other disorders associated with neurofilament aggregation, the neurofilaments and their building blocks could be dysregulated reminiscent of NFL loss in axons. In both situations, axonal neurofilament stoichiometry and turnover are disrupted (Stone et al., 2019). Our study provides some suggestions on what pathways are altered in the axons in NFL loss: increased movement of mitochondria, decreased amplitude of mEPSCs and reduced axonal caliber. It is paradoxical that organelle movements are decreased by the NFL missense mutant (Van Lent et al., 2021) and increased by the nonsense mutant studied here, yet both of these mutations result in similar CMT phenotypes. Hypothetically, reduced organelle movement may hinder the delivery of organelles to the sites where they are needed, such as synapses or nodal regions, whereas increased movements may decrease their ability to stay there. Thus, both missense and nonsense mutations could have the same end result on organelle positioning and cause local bioenergetics deficiency at key locations in the axons. Future studies on CMT disease mechanisms could compare motor neurons with *NEFL* nonsense and missense mutations to validate common pathological pathways that converge in axonal degeneration. Furthermore, it would be important to explore the elastic properties of axons in NFL-linked CMT to rule out the possibility of loss of structural support by neurofilament dysregulation (Grevesse et al., 2015) as the axons with dysregulated neurofilaments may be structurally more prone to damage caused by movements of the body (Gafson et al., 2020).

In summary, iPSC-MN provide a promising model for studying the pathological mechanisms of NFL in human disease. Detailed characterization of mutant MN in comparison to isogenic controls allows to identify targetable mutation-specific alterations. For the development of treatments for CMT, it is critical to identify the final disease pathways common to different *NEFL* mutations as well as to mutations in other CMT disease genes.

## Materials and Methods

### iPSC Culture

Human fibroblasts from patients (Sainio et al., 2018) and healthy controls (Trokovic et al., 2015) were reprogrammed into pluripotent stem cells with overexpression of *MYC*, *KLF4*, *SOX2*, and *OCT4* by the Biomedicum Stem Cell Center (University of Helsinki, Finland) as in Trokovic et al. (2015). Stem cells were grown in feeder-free conditions with Matrigel (Corning) and E8 with E8 supplement (Gibco) in normoxia at 37°C. At 90% confluency, cells were passaged with 0.5 mM EDTA (Invitrogen) in PBS.

### Genome Editing of iPSC

*NEFL* KO iPSC were generated with CRISPR-SpCas9 technology as described before (Saarimäki-Vire et al., 2017). Briefly, KO guideRNA (gRNAs) sequences, targeting 5′UTR and the first exon as in Harjuhaahto et al. (2020) were designed in Benchling (https://benchling.com). Transcriptional units for gRNA expression were prepared by PCR amplification (Balboa et al., 2015). Detailed protocol is provided by Addgene (http://www.addgene.org/78311/). GuideRNA pairs were tested by co-transfection with CAG-Cas9-T2A-EGFP-ires-puro plasmid (Addgene plasmid #78311) into HEK293 cells. The genomic region flanking gRNA target sites was amplified and editing efficiency was detected by agarose gel electrophoresis.

5′UTR (TGC​GAT​CGA​TCA​CGG​CAC​GC) and exon 1 (CGG​CTG​ATG​GAA​GCG​CGC​AA) sequences were used for the CRISPR-SpCas9 targeting of *NEFL*. Three μg of endotoxin-free CAG-Cas9-T2A-EGFP-ires-puro plasmid and 500 ng of each gRNA were electroporated into 2 million control iPSCs with the Neon Transfection System (1100 V, 20 ms, two pulses, Thermo Fisher Scientific). Electroporated cells were transferred to prewarmed Matrigel-coated plates containing E8-medium with 10 μM ROCK inhibitor (ROCKi) (Y-27632 2HCl, Selleckchem). GFP positive live iPSC were sorted with BDInflux Flow Cytometer at the Biomedicum Flow Cytometry Unit (University of Helsinki, Finland) into 96-well plates with Matrigel coating and 5 μM ROCKi and CloneR™ (StemCell Technologies) E8 media. Expandable iPSC colonies were screened by PCR, sequencing, and Western blotting.

### Karyotype

iPSCs at 40%–70% confluency were treated with KaryoMax Colecemid (10 μg/ml, Thermo Fisher) for 4 h to arrest cells in metaphase. The cells were then dissociated with Tryple Select (Thermo Fisher) and centrifuged at 200 g 5 min. The dissociated cells were treated with 75 mM KCl (Acros Organics) for 10 min at 37°C and centrifuged again as above. Finally, the cells were fixed with serial incubations (3 × 3 ml followed with 200 g 5 min centrifugation) of 25% acetic acid (Thermo Fisher) in methanol (Honeywell) and imaged for karyotyping.

### Motor Neuron Differentiation

To differentiate iPSCs into motor neurons, we used a suspension-based protocol by Maury et al. (2015) and Guo et al. (2017) with slight modifications. MN differentiation base media (MNb) containing DMEM/F12 (Gibco), Neurobasal (Gibco) 1:1, 0.5× N2 (Life Technologies), 0.5× B27 (Life technologies), L-ascorbic acid 0.1 mM (Santa Cruz) and Primocin 100 μg/ml (Invivogen) was used throughout the differentiation. Briefly, on day 0, iPSC were dissociated with 0.5 mM EDTA (Invitrogen) into small clusters and resuspended into ultralow attachment plates (Corning) with differentiation media 1; MNb supplemented with 40 μM SB431542 (Merck), 0.2 μM LDN-193189 (Merck/Sigma), 3 µM CHIR99021 (Selleckchem), and 5 µM Y-27632 (Selleckchem). The following day (day 1), differentiation media 1 was changed. On day 2, differentiation media 1 was changed to differentiation media 2; MNb supplemented with 0.1 µM retinoic acid (RA) (Fisher) and 0.5 µM smoothened agonist (SAG) (Calbiochem). Media 2 was changed on day 4. On day 7, differentiation media 2 was changed to differentiation media 3; MNb supplemented with 0.1 µM RA (Fisher), 0.5 µM SAG (Calbiochem), BDNF (10 ng/ml, Peprotech), and GDNF (10 ng/ml, Peprotech). On day 9 differentiation media 3 was changed to media 4; MNb supplemented with 0.1 µM RA (Fisher), 0.5 µM SAG (Calbiochem), BDNF (10 ng/ml, Peprotech), GDNF (10 ng/ml, Peprotech), and 20 µM DAPT (Calbiochem). Motor neuron progenitor spheroids were dissociated into single cells for plating on day 10 by using Accumax (Invitrogen). Dissociated motor neuron progenitors were then plated on poly-D-lysine 50 μg/ml (Merck Millipore) and laminin 10 μg/ml (Sigma-Aldrich) coated plates in differentiation media 4 additionally supplemented with 10 μM ROCKi (Sellechem). Half of media 4 was changed on day 11 (no ROCKi added). At day 14, half of media 4 was changed to media 5; MNb supplemented with BDNF (10 ng/ml, Peprotech), GDNF (10 ng/ml, Peprotech), and 20 µM DAPT (Calbiochem). At day 16 half of media 5 was changed to media 6; MNb supplemented with BDNF (10 ng/ml, Peprotech), GDNF (10 ng/ml, Peprotech), CNTF (10 ng/ml, Peprotech), and 20 µM DAPT (Calbiochem). From day 18 onward, the cells were switched to motor neuron maturation medium; MNb supplemented with BDNF, GDNF, and CNTF (each 10 ng/ml, Peprotech). Media were changed every other day during the first week of maturation, days 18 to 25, and twice a week afterward by replacing half of the medium.

### SDS-Page Western Blotting

Proteins from day 35 of MN differentiation were harvested with 1× RIPA-buffer + HALT™ protease inhibitor cocktail (Thermo Fisher) according to the manufacturer’s instructions. Protein lysates were mixed with 6×SDS-loading buffer (Thermo Fisher) containing β-mercaptoethanol (Bio-Rad) and boiled for 5 min. Then, 5 µg of protein were run on TGX-stain-free acrylamide gels and transferred to 0.2 µm Nitrocellulose-membrane with the TransBlot Turbo transfer system (all from Bio-Rad). Membranes were blocked with 5%-nonfat dry milk (Valio) in Tris-base-solution with Tween20 0.1% (TBS-T) and incubated in primary antibodies with 3%-BSA (BioWest) TBS-T. Primary antibodies used in immunoblotting: mouse mAb NFL N-terminal 1:1000 (sc-390732, Santa Cruz), mouse mAb NFL 1:1000 (N5139, Sigma), rabbit mAb NFL C-terminal 1:1000 (MA5-14981, Thermo Fisher), rabbit pAb NFM 1:1000 (20664-1-AP, Proteintech), rabbit pAb NFH 1:1000 (pan-NFH, ab8135, Abcam), mouse mAb TUJ1 1:1000 (801,201, Biosite), rabbit pAb PRPH 1:1000 (AB1530, Merck), and mouse mAb INA 1:1000 (MAB5224, Merck) and secondary antibodies: Goat-anti-mouse (Jackson) 1:5000 or Goat-anti-rabbit (Jackson) 1:10,000. Secondary antibody signal was detected with an ECL reagent (Western Lightning, Perkin Elmer). Membranes were imaged with Chemidoc XRS+ (Bio-Rad).

In neurofilament oligomer WB, protein lysates were not treated with β-mercaptoethanol and were not boiled to prevent neurofilament oligomer dissociation. Otherwise, WB was performed as above.

To confirm the absence of insoluble truncated NFL in KO and PT neurons, the pellets, after protein extraction with RIPA-buffer, were treated with undiluted 6×SDS-buffer (Thermo Fisher) containing B-mercaptoethanol (Bio-Rad) and boiled at 98°C for 10 min. The whole sample was loaded onto a gel and WB continued as above.

In filament analysis during differentiation, samples were collected during differentiation, on indicated days (Figure 1F) and processed for WB as above.

### Immunocytochemistry

Days 32–35, motor neurons on cover glasses were fixed with 4% paraformaldehyde (PFA) for 20 min in room temperature and then permeabilized with phosphate buffer saline (PBS) 0.2% Triton ×100 (Fisher) for 15 min. The samples were blocked with protease-free 5% BSA (Jackson ImmunoResearch) in PBS 0.1% Tween20 (PBS-T) for 2 h in RT. The following primary antibodies were incubated overnight at 4°C: mouse mAb NFL N-terminal 1:1000 (sc-390732, Santa Cruz), rabbit mAb NFL C-terminal 1:1000 (MA5-14981, Thermo Fisher), rabbit pAb NFM 1:500 (20664-1-AP, Proteintech), mouse mAb NFH non-phosphorylared 1:500 (SMI32, 801701, Biolegend), rabbit mAb NFH (pan-NFH, ab8135, Abcam) mouse mAb TUJ1 1:1000 (801201, Biosite), rabbit pAb PRPH 1:1000 (AB1530, Merck), mouse mAb INA 1:1000 (MAB5224, Merck), and mouse mAb ISL-1 1:50 (40.2D6, DSHB). The following secondary antibodies were used: Alexa Fluorophore (Thermo Fisher) goat-anti-mouse 488/594 and goat-anti rabbit 488/594. Cover glasses were applied on slides with Vectashield DAPI (Vectorlabs) and imaged with Axio Observer Z1 (Zeiss) or Andor Dragonfly spinning disk confocal (Oxford instruments).

iPSCs were stained as above at 50% confluency with rabbit Ab Nanog 1:1000 (4903, Cell signaling) and mouse Ab 1:100 Tra1-60 (MA1-023, Invitrogen) antibodies.

Differentiation efficiency was determined from DAPI, ISL1/2, TUJ1, and NFM stained samples on day 35 of differentiation. First, the DAPI staining was used to determine cell viability in fixed samples: evenly DAPI stained nuclei were determined to be live during fixation and DAPI signal from bright spots was determined unspecific or originating from dead cells. Second, the ratio of ISL1/2 and TUJ1/NEFM positive cells was determined by calculating evenly DAPI and ISL1/2 stained cell nuclei or TUJ1/NEFM stained cell cytoplasm with ImageJ package Fiji Cell Counter plugin in six replicates from three independent differentiations with a total of 18 micrograph frames.

### Microfluidics

Motor neurons, 150,000 cells per device on day 10 of differentiation, were plated on Xona 450 µm microfluidic devices (Xona microfluidics) coated with poly-D-lysine 50 μg/ml (Merck Millipore) and laminin 40 μg/ml (Sigma-Aldrich). Pre-assembled devices were used for axon growth and axotomy experiments, and polydimethylsiloxane (PDMS) devices mounted on coverslips for the organelle tracking assay.

Axon growth and regrowth after axotomy was assayed by phase-contrast imaging of the axonal compartment daily from differentiation days 15–24. Mechanical axotomy was performed with forceful aspiration on the axonal side on day 22.

Phase-contrast images were analyzed for axonal coverage in Fiji ImageJ by thresholding and measuring the area of signal in the axonal compartment. To overcome variation between differentiations, axonal area was normalized within the experiment to the average signal in all cell lines on day 15. Additionally, the thresholded images were skeletonized and assayed with the Fiji ImageJ Sholl plugin to measure axonal branching.

### Transmission Electron Microscopy

Day 35 motor neuronal cultures on cover glasses were fixed with 2% glutaraldehyde (EMS) in 0.1 M NaCac buffer for 30 min at room temperature. The fixative was then washed with NaCac buffer. Fixed neurons were prepared according to standard protocols for transmission electron microscopy by Electron Microscopy Unit of the Institute of Biotechnology, University of Helsinki. Briefly, for postfixation, the glutaraldehyde coverslips were incubated for 1 h in 0.1 M NaCac buffer with OsO4 (1%) and potassiumferrocyanide (15 mg/ml). Postfixed samples were then washed with 0.1 M NaCac buffer, dehydrated with a graded ethanol series (70%, 96%, 100%) and dipped in acetone. Epon resin in a beam capsule was applied on top of an area with axons on the coverslips and incubated for 2 h before baking at 60°C for 14 h. The polymerized resin was removed from the coverslip, and sections were cut with a diamond knife for TEM. Ultrathin sections were observed with Jeol 1400 (Jeol) transmission electron microscope at 80,000 V. ImageJ package Fiji (Schindelin et al., 2012) was used to assess axonal parameters.

### Quantitative Real-Time PCR

RNA was extracted from day 35 differentiated motor neurons with the NucleoSpin RNA kit (Macherey-Nagel). cDNA was reverse-transcribed with Maxima first strand cDNA synthesis kit (Thermo Fisher). Transcript levels were analyzed by qPCR amplification in CFX Real-time system C1000Touch (Bio-Rad) with SYBR-green Flash (Thermo Fisher) by gene-specific primers. Primers used: *GAPDH* (Forward: CGC​TCT​CTG​CTC​CTC​CTG​TT, reverse: CCA​TGG​TGT​CTG​AGC​GAT​GT), *TUBB3* (Forward: GCC​AAG​TTC​TGG​GAA​GTC​AT, reverse: CCA​CTC​TGA​CCA​AAG​ATG​AA), *NEFL* (Forward: CAA​GAC​CCT​GGA​AAT​CGA​AG, reverse: TGA​AAC​TGA​GTC​GGG​TCT​CC), *B-actin* (Forward: CCT​GGC​ACC​CAG​CAC​AAT, reverse: GGG​CCG​GAC​TCG​TCA​TAC), *NEFM* (Forward: TGC​AGT​CCA​AGA​GCA​TCG​AGC, reverse: AGT​CTC​TTC​ACC​CTC​CAG​GAG​TT), and *NEFH* (Forward: AGT​CCG​AGG​AGT​GGT​TCC​GA, reverse: GTC​CAG​CTG​CTG​AAT​GGC​TTC).

### Electrophysiology

Whole-cell patch-clamp recordings were performed as in Harjuhaahto et al., 2020 (Harjuhaahto et al., 2020) on motor neurons after day 35 of differentiation. In brief, coverslips with cultured neurons were placed in the recording chamber on the visually guided patch-clamp setup, perfused with ACSF of the following composition (mM): 124 mM NaCl, 3 mM KCl, 1.25 mM NaH_2_PO_4_, 1 mM MgSO_4_, 26 mM NaHCO_3_, 2 mM CaCl_2_, 15 mM glucose, and 95% O_2_/5% CO_2_, 30°C. For recording of passive membrane properties and action potential firing, cells were patched with microelectrodes containing the intracellular solution, which included (mM): 105 K gluconate, 15 KCl, 5.3 CaCl_2_, 5 NaCl, 10 HEPES, 10 EGTA, 4 MgATP, 0.5 Na_2_GTP, pH 7.2. Series resistance, input resistance, and capacitance were recorded in voltage clamp mode at −70 mV by injection of 5 mV voltage steps. Resting membrane potential and spontaneous activity were recorded in current clamp mode without any background current injected. For recording of the evoked action potentials, baseline membrane potential was held at −60 mV, and depolarizing current steps with the increasing increment of 10 pA were injected for 1 s. mEPSCs were recorded in a voltage clamp mode at −70 mV in ACSF containing tetrodotoxin (TTX, 1 μM) and picrotoxin (100 μM). Intracellular solution contained (in mM): 110 CsMeSO_3_, 5.3 CaCl_2_, 5 NaCl, 10 HEPES, 10 EGTA, 5 lidocaine N-ethyl chloride, 4 MgATP, 0.5 Na_2_ATP. Data acquisition and analysis were done as described in (Harjuhaahto et al., 2020).

### Organelle Movement

Day 30 motor neurons grown in Xona PDMS (Xona microfluidics) devices were incubated with Mitotracker Green FM 50 nM (Thermo Fisher) and Lysotracker Red DND-99 50 nM (Thermo Fisher) for 1 h. Media was changed and axonal organelle movement videos captured with Andor Dragonfly (Oxford instruments) spinning disk confocal; pinhole 40, 1 min capture, 1 frame/s. Movies were analyzed as videos (all moving organelles per video) and as kymographs from individual axons.

The movie processing was performed in Fiji (Schindelin et al., 2012), and Trackmate-plugin (Tinevez et al., 2017) was used for video analysis. The following steps were followed in video analysis: enhance contrast to 5% (use stack histogram, apply to all frames), convert to 8 bit, Difference tracker-plugin (Andrews et al., 2010) (max distance 3 and max gap 3), median filter (2 px), Trackmate-plugin DoG detector (estimated blob diameter 2 and threshold 1), linear motion LAP tracker (2,2,2) and track filtering (displacement >1 px and duration >2 frames). Total displacement of all organelles per video was normalized to total count of organelles. Organelle count was estimated with the Fiji Analyze particles function from the thresholded 8-bit contrast-enhanced first frame of the movie after median filter (2 px). Particles were calculated as separate organelles when their area was between 0.1 and 2 μm^2^.

For kymograph analysis, sections of individual axons (50–200 μm) with at least one clearly moving organelle were analyzed with the Kymoanalyzer-plugin (v1.01) according to plugin instructions (Neumann et al., 2017). Moving organelles with less than 0.2 μm of total displacement were termed as dynamic pausing.

### Treatments

Differentiated patient motor neurons were treated with gentamicin 50–500 μg/ml (Gibco), PTC-124 25-200 μM (Merck Calbiochem) and amlexanox 25–250 μM (Merck). Amlexanox and PTC-124 were diluted in DMSO as in manufacturer’s instructions and gentamicin solution was added directly to cells. To achieve a proper treatment window, neurons were treated starting on different days (days 14–16) and treated from 7 to 12 days. Neuron health and neurite disruption were monitored during the treatments daily with phase-contrast microscopy.

### Simoa

NFL was measured from patient serum as in (Järvilehto et al., 2021) with the same controls.

In the cell experiments, 1 ml of media was collected from day 30 iPSC-MN. Media were centrifuged at 13,000 g for 5 min and frozen. Media were thawed on ice, mixed, centrifuged at 10,000 g 5 min and diluted (1:50) to measure NFL concentration on a 96-well format in duplicate wells with the Single molecule array (Simoa) HD-1 analyzer (Quanterix, Billerica, MA) and the NF-Light Advantage Kit (ref. 103186) according to manufacturer’s instructions (Quanterix, Billerica, MA). Vincristine (0.5 μM, Sigma-Aldrich) was applied to iPSC-MN for 24 h as a positive control.

### Statistics

Graphpad Prism (Graphpad Software version 9) was used for statistical analysis. One-way ANOVA with Dunnett’s multiple comparisons to CTR was used to assess statistical differences between CTR and other cell lines. Two-way repeated-measures ANOVA or Two-way ANOVA with Dunnett’s multiple comparison was used in comparisons with more than one variable, unless otherwise indicated. Fisher’s exact was used to compare proportions of axons containing neurofilaments. A *p*-value of less than .05 was termed as significant.

## Data Availability

The original contributions presented in the study are included in the article/Supplementary Material, further inquiries can be directed to the corresponding author.
