# Supplementary figures and images for: Neurofilament Light Regulates Axon Caliber, Synaptic Activity, and Organelle Trafficking in Cultured Human Motor Neurons
Source: Front Cell Dev Biol. 2022 Feb 14;9:820105. doi: 10.3389/fcell.2021.820105 (PMC8883324; doi:10.3389/fcell.2021.820105)

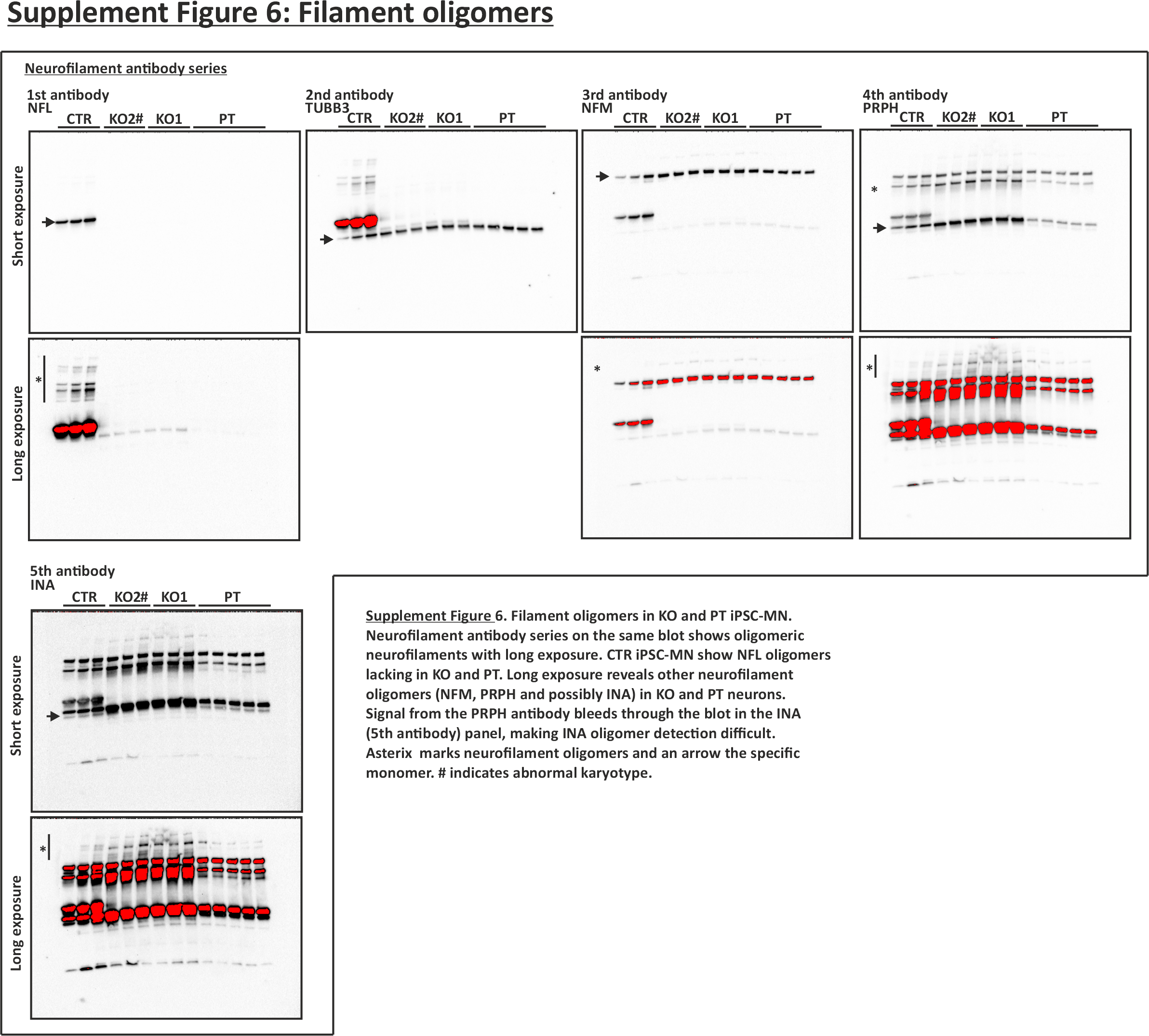

Supplement: Supplementary file 1 [file Image6.TIF]

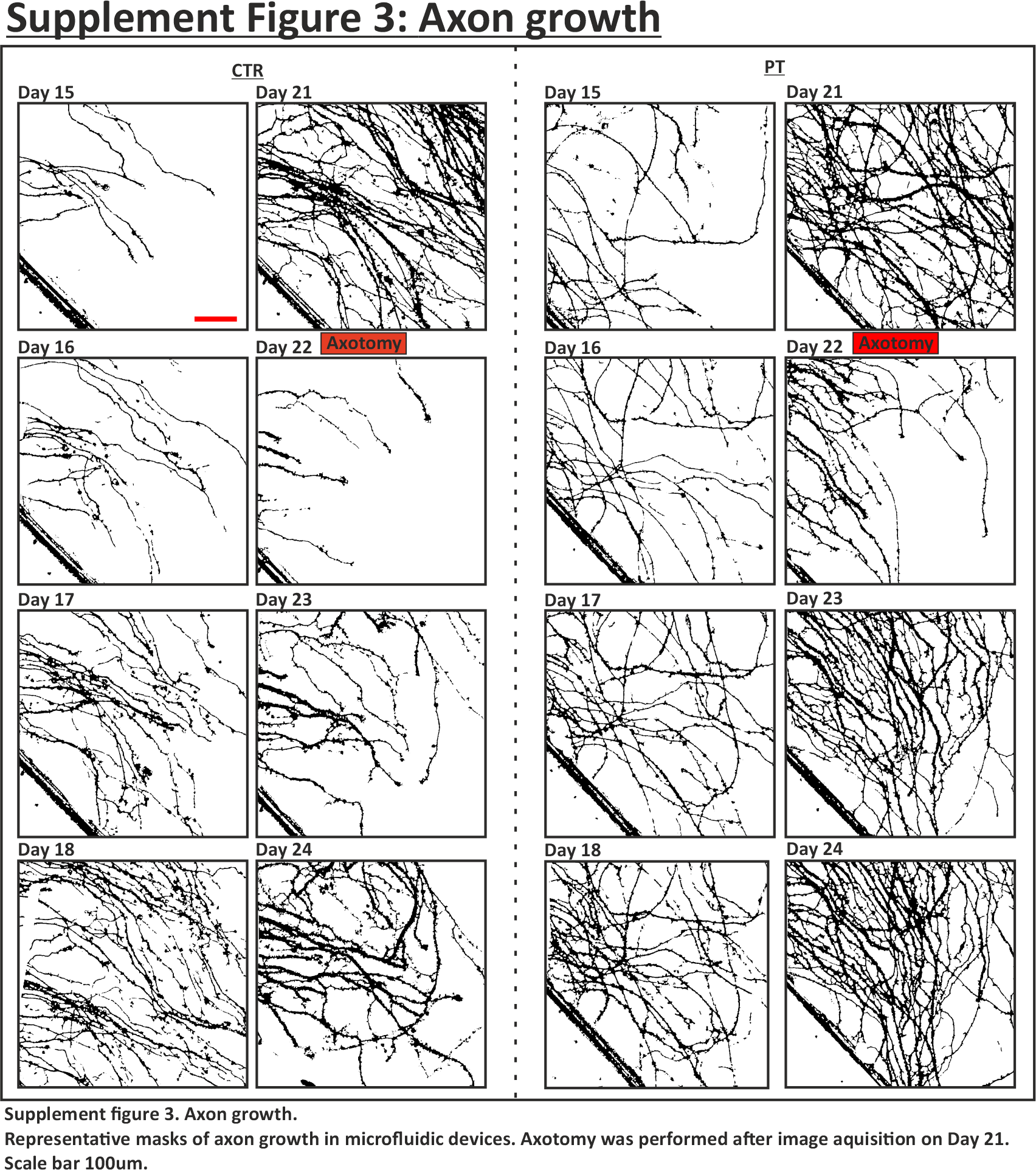

Supplement: Supplementary file 2 [file Image3.TIF]

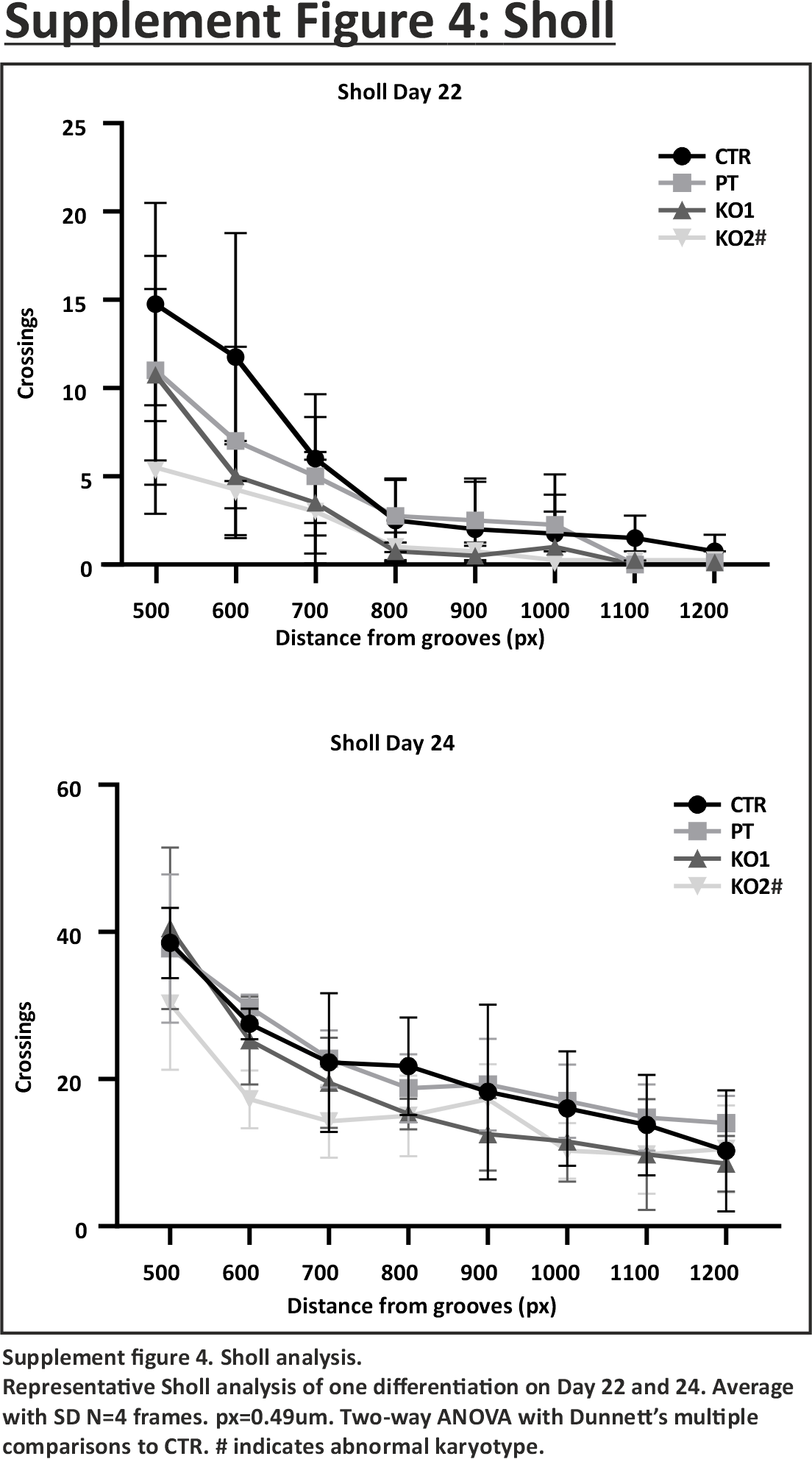

Supplement: Supplementary file 3 [file Image4.TIF]

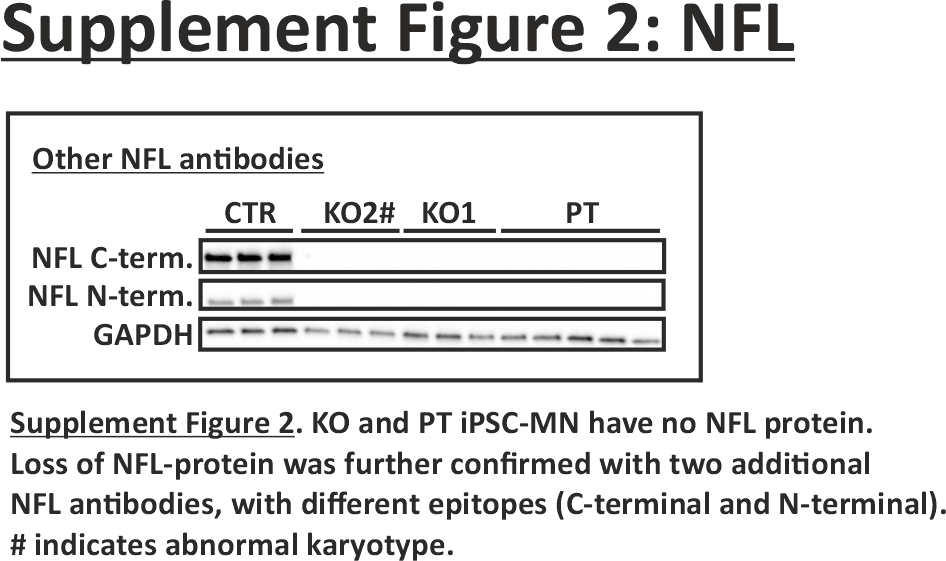

Supplement: Supplementary file 4 [file Image2.TIF]

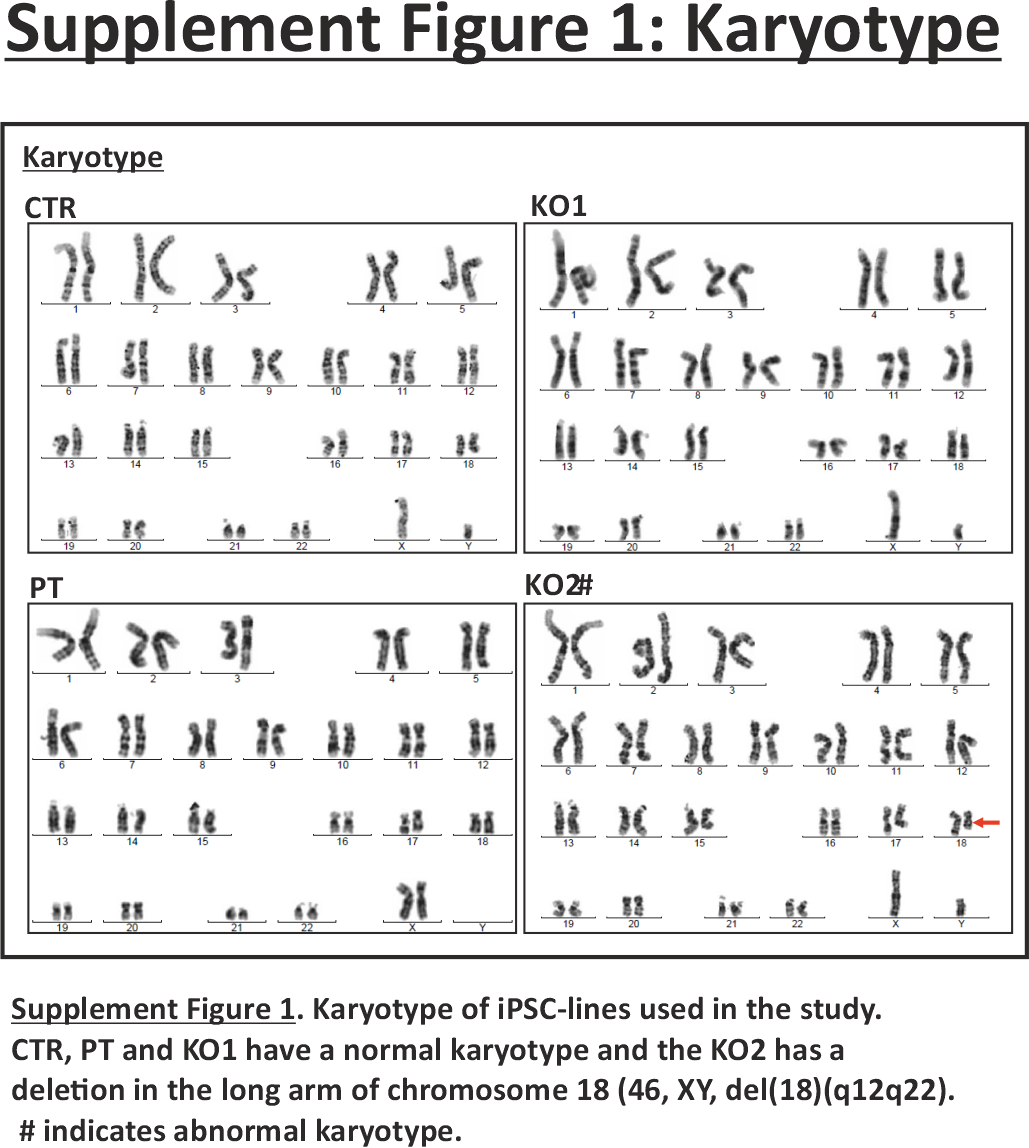

Supplement: Supplementary file 5 [file Image1.TIF]

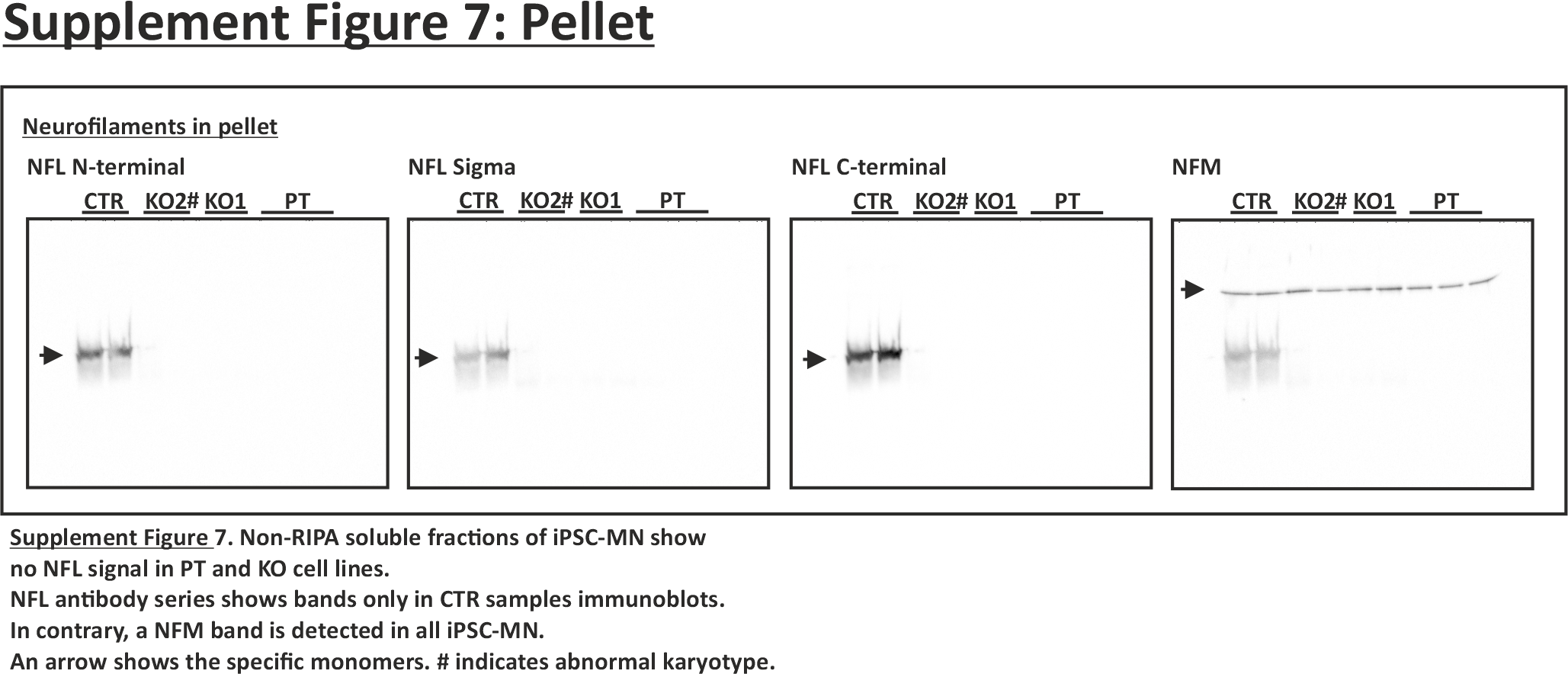

Supplement: Supplementary file 6 [file Image7.TIF]

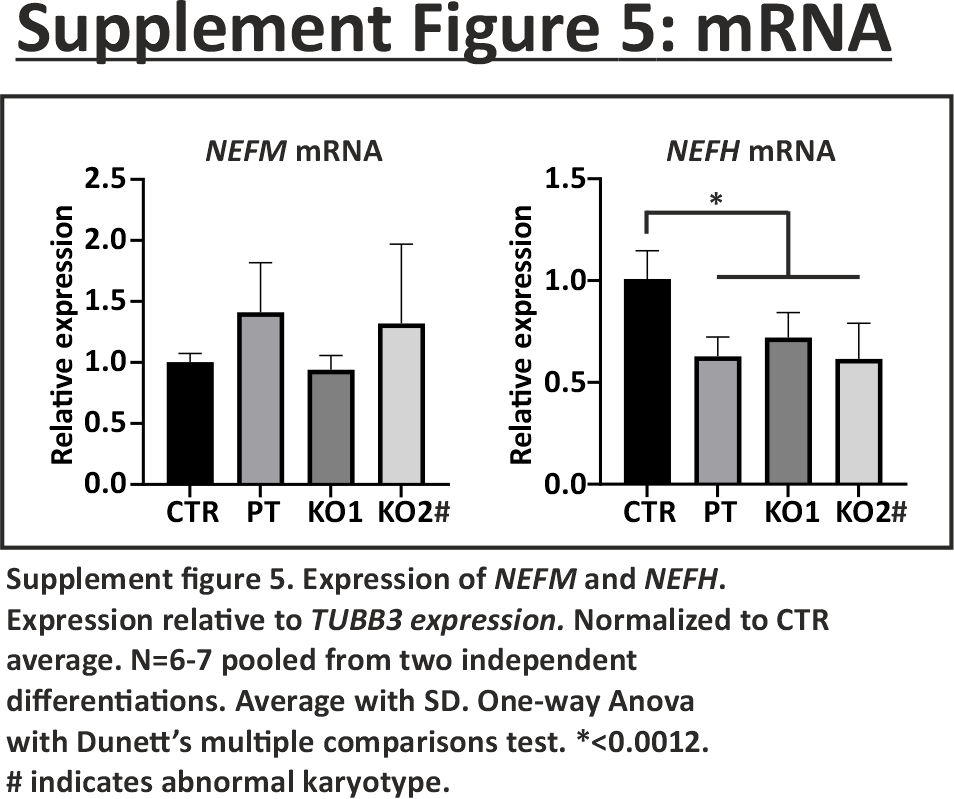

Supplement: Supplementary file 7 [file Image5.TIF]
